# Concomitant Pulmonary and Biliary Manifestations of Ulcerative Colitis: A Case Report

**DOI:** 10.1002/ccr3.70125

**Published:** 2025-01-16

**Authors:** Pritee Shrestha, Philip Shara, Babajide Adio

**Affiliations:** ^1^ Internal Medicine MercyOne North Iowa Medical Center Mason City Iowa USA; ^2^ Medical College of Wisconsin Milwaukee Wisconsin USA

**Keywords:** cryptogenic organizing pneumonia, interstitial lung diseases, primary sclerosing cholangitis, ulcerative colitis

## Abstract

Ulcerative colitis can present with extra‐intestinal manifestations, including interstitial lung disease and primary sclerosing cholangitis. When pulmonary symptoms precede gastrointestinal, diagnosis can be challenging. Consideration of Ulcerative colitis in patients with unexplained lung and hepatic pathology is crucial, as a failure of timely intervention can lead to multiorgan complications.

## 1 Introduction

Extra gastrointestinal complications of ulcerative colitis (UC) have been well documented, although concomitant biliary and lung parenchymal disease remains uncommon. Pulmonary manifestations of UC comprise various pathologies, cryptogenic organizing pneumonia being the most common [1]. The risk of Primary Sclerosing Cholangitis (PSC) in the setting of preexisting UC has been well studied. We describe diagnostic complexity in a 48‐year‐old female, initially suspected to have vasculitis due to her pulmonary presentations, who was later discovered to have UC with contaminant PSC. Collectively as well as individually, these conditions are associated with high morbidity and mortality. PSC is associated with high‐risk hepatocellular and biliary carcinoma. We aim to highlight the diagnostic complexity posed due to the atypical presentation of UC in our patients and aid providers in the management of IBD‐associated life‐threatening intra and extra‐gastrointestinal complications.

## 2 Case History/Examination/Presentation

A 48‐year‐old female was hospitalized for productive cough, congestion, and shortness of breath (SOB). Her past medical history was positive for weight loss and ongoing pneumonia. She was initially suspected of having community‐acquired pneumonia and was treated with antibiotics. Two weeks later, she was hospitalized again for hematemesis and melena. Associated symptoms included fever, night sweats, chills, and fatigue. The patient denied significant travel/occupational history. Physical examination revealed no apparent distress, regular rate and rhythm, without murmurs, crackles at the bases bilaterally, the abdomen was soft, non‐distended, and non‐tender to palpation. Bowel sounds were present in all four quadrants, no clubbing, cyanosis, or edema in extremities, no rashes, bruises, or skin lesions seen. Vitals were stable and patient was saturating above 90% in 2 L NC.

**FIGURE 1:**
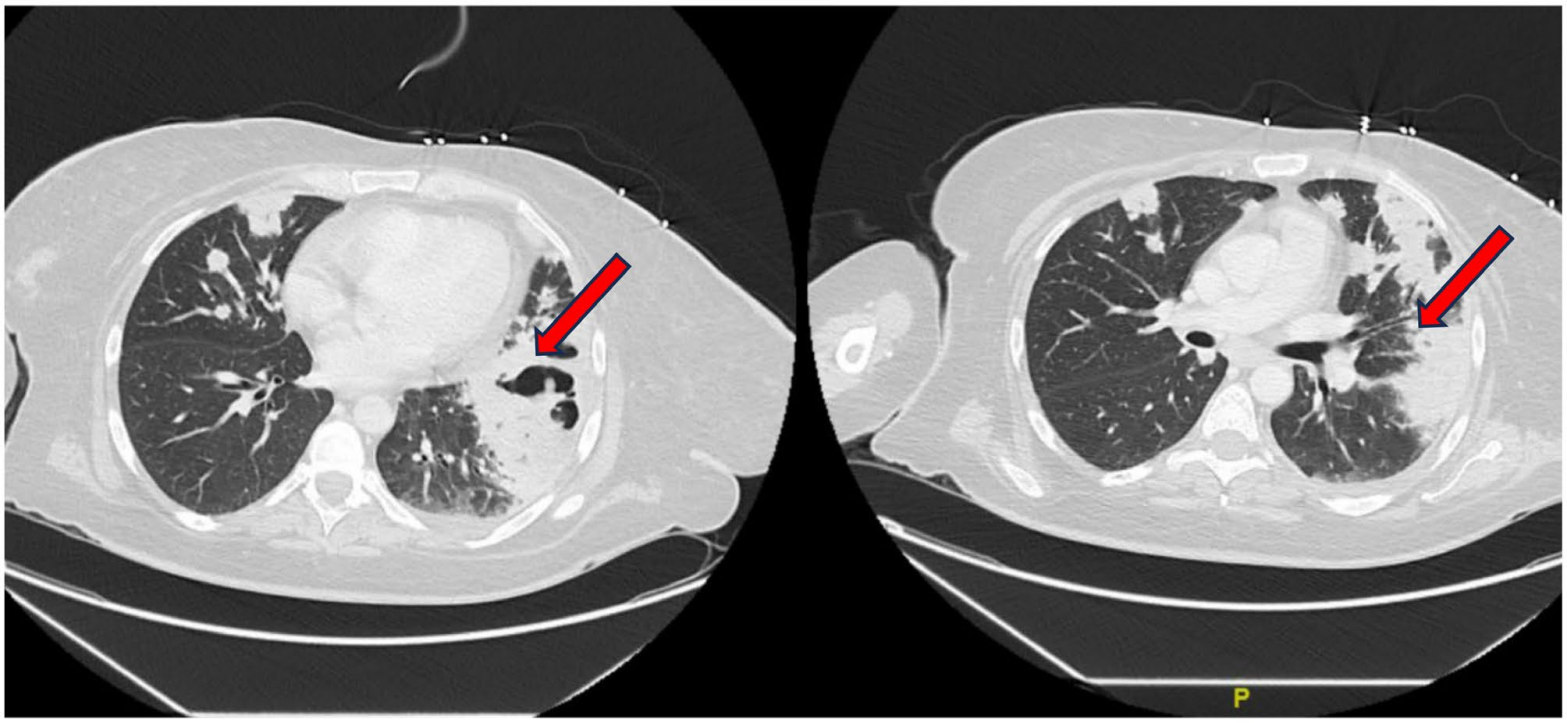
Initial computed tomography (CT) of the chest 07/2019: Extensive bilateral pulmonary consolidation, cavitary and noncavitary consolidative nodules, and suspected peripheral left lung base abscess, some pleural effusion.

Investigations initially began with basic laboratory tests. The patient was found to have a WBC of 46.6 × 103/u, hemoglobin 8.3 g/dL, lactate 3.1 mMol/L, INR 2.7, and fecal occult blood was positive. Computed tomography (CT) of the chest showed extensive bilateral pulmonary consolidations with thick‐walled cavitary/non‐cavitary lesions and a left lower lung abscess suspicious of vasculitis (Figure 1). BAL was negative for infectious etiology. GMS and AFB stains were negative for fungal or acid‐fast organisms. Tuberculosis and hepatitis workups were negative. The left lower lung core needle biopsy section showed a portion of benign lung tissue involved by occasional interalveolar fibroblastic/myofibroblast plugs consistent with organizing granuloma. A pancytokeratin immunostaining highlighted the typical alveolar architecture without abnormal epithelial proliferation. Esophagogastroduodenoscopy (EGD) was performed. EGD showed friable gastric mucosa and gastritis. A gastric biopsy showed acute inflammation with ulcerations and was negative for *Helicobacter Pylori* .

## 3 Methods (Differential Diagnosis, Investigations, and Treatment)

Differential diagnosis at this time included unresolving bacterial pneumonia, interstitial lung disease, vasculitis, or autoimmune disease. It was unclear at this time if the patient's GI presentation including melena and associated EGD findings were associated disorders. Broad treatment regime encompassing her multiple acute comorbidities were initiated. She was started on IV pantoprazole, IV Vancomycin, Meropenem, and Fluconazole for empiric coverage. The patient improved slowly and was eventually transitioned to oral antibiotics upon discharge. The decision to continue clindamycin and sulfamethoxazole/trimethoprim outpatient was made per infectious disease evaluation given the significantly elevated WBC count on presentation, which continued to decrease on antimicrobial therapy despite multiple negative cultures as well as due to the lack of definitive diagnosis at that time. This regime would target anaerobic bacteria and gram‐positive/negative respiratory pathogens, providing continued coverage for polymicrobial infection, aspiration pneumonia, and community‐acquired pneumonia.

Outpatient follow‐up evaluation by infectious disease revealed that the patient had continued SOB and constitutional symptoms. X‐ray showed worsening consolidation on the right side, and she was thus referred to pulmonology for assessment. Serology obtained at this time due to concern for vasculitis showed positive proteinase‐3 antibodies, eosinophilia, elevated alkaline phosphatase (ALKp) at 395, and ANA of 1: 640. ESR 74, and CRP 4.6. Suspicion for vasculitis remained considerably high at this point; prednisone taper therapy was initiated.

**FIGURE 2:**
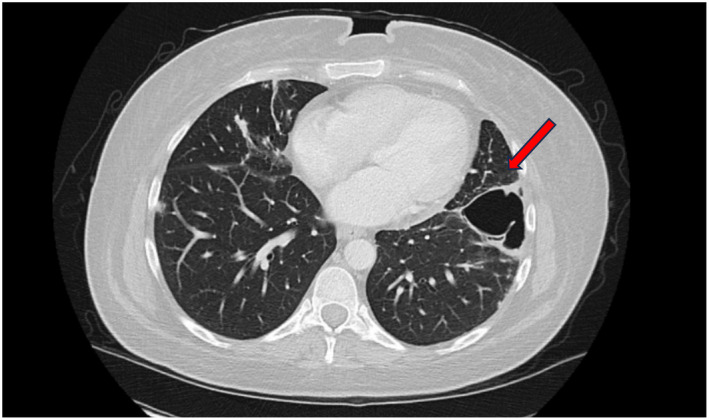
Chest CT 09/2019: Patchy areas of consolidation/nodules bilaterally improved since prior examination likely from steroid treatment. Continued cavitary within LEFT lower lobe like prior examination.

**FIGURE 3:**
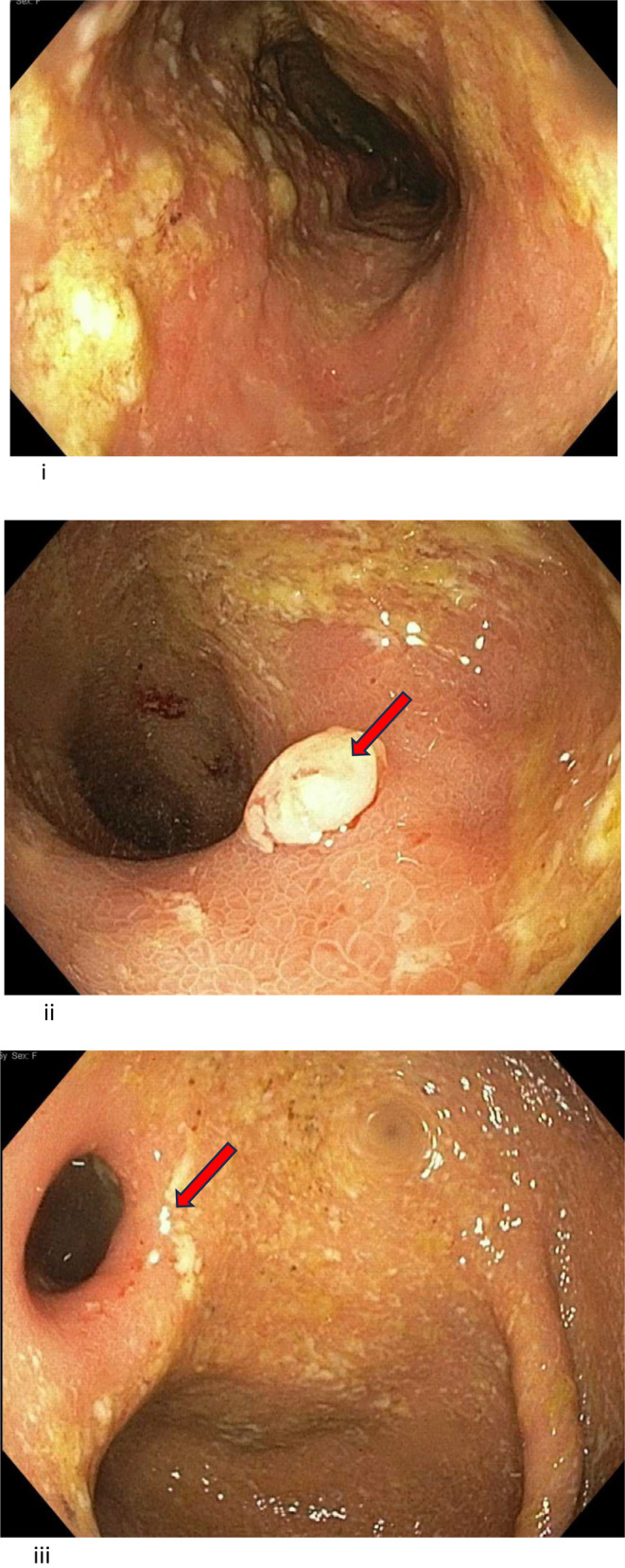
Colonoscopy 10/2019: Diffuse granularity in the ileum without ulcers, diffuse inflammation as well as friability in the entire colon more prominent in the right side and aphthous Ileocolic valve and evidence of ileitis. (i) Ascending colon with diffuse inflammation and friability. (ii) Colonic Pseudo polyps. (iii) Aphthous Ileocolic valve and evidence of ileitis.

**FIGURE 4:**
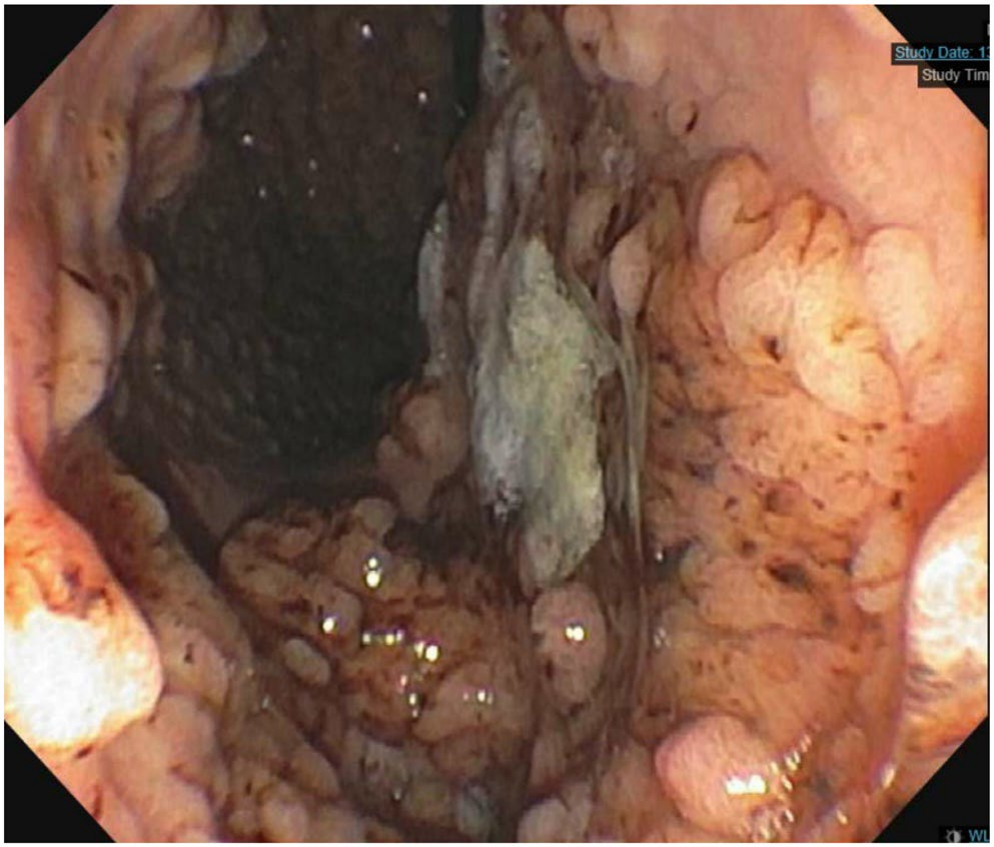
ERCP showing marked bile duct inflammation.

Repeat chest CT showed marked improvement of multifocal nodular opacities. (Figure 2). Other new laboratory follow‐up studies revealed elevated IgG at 1990 mg/DL and Alk‐p increased to 400. Colonoscopy at this time revealed diffuse inflammation of the colon, more prominent on the right side, characterized by altered vascularity, congestion, erythema, friability, granularity, and mucus found in the entire colon. (Figure 3i–iii). Biopsy revealed active chronic colitis without any granulomas or dysplasia. CMV immunostaining was negative. She subsequently had an MRI of the abdomen with MRCP without and with IV contrast. This showed diffuse multifocal structuring of the intrahepatic biliary tree, given her predominantly right‐sided colitis in the setting of elevated ALK‐phosphatase and the possibility of PSC. Subsequent Magnetic Resonance cholangiopancreatography (MRCP) was performed, which showed diffuse multifocal strictures of the intrahepatic biliary tree. A blood test was negative for alpha‐fetoprotein, and CA 19–9 of 76, and the IgG 4 level was mildly elevated at 172. Fibro‐scan showed advanced liver fibrosis. Follow‐up Endoscopic Retrograde Cholangiopancreatography (ERCP) revealed evident biliary duct inflammation (Figure 4) and widespread strictures. The entire extrahepatic duct was balloon dilated, and samples were sent for biopsy, which showed abnormal glandular cells favoring reactive/degenerative processes without dysplasia and biliary type mucosa with acute on chronic inflammation. The IgG4/IgG ratio was < 40%, ruling out igG4‐related pathology. The total constellation of these follow‐up studies led to the final diagnosis of ulcerative colitis with associated PSC. Due to the impressive inflammatory changes, the decision was made to treat aggressively with Infliximab instead of Methyl‐prednisone monotherapy.

## 4 Outcome and Follow‐Up

The patient continued outpatient infliximab infusions with subsequent remission of her ulcerative colitis as well as her pulmonary symptoms. The patient was recommended a repeat liver function test every 3 months, contrast imaging of the liver every 6 months to screen for primary liver cell cancer, and MRCP once a year surveillance for any changes in bowel duct anatomy along with a recheck of the CA 19–9 bile duct cancer marker. Overall, despite a prolonged and complicated hospital course, the patient was successfully managed for her multiple complex comorbidities.

## 5 Discussion

Lung parenchymal involvement in UC is relatively uncommon. Pulmonary manifestations of UC comprise various pathologies and cryptogenic organizing pneumonia being one of the common manifestations [1]. Histological evaluation of affected pulmonary lesions can reveal pulmonary infiltrates, eosinophilia, and bronchiolitis obliterans [2]. Other lung parenchymal involvement of UC includes conditions like interstitial pneumonitis, bronchiolitis, eosinophilic pneumonia, bronchiolitis obliterans organizing pneumonia, fibrosis, Langerhans's cell histiocytosis, and vasculitis [1]. Shared embryonic origin seems to be the primary mechanism by which patients with IBD have pulmonary involvement, as columnar epithelium, goblet cells, and submucosal lymphoid tissue are present in both gastrointestinal and pulmonary epithelial tissues. Patients with IBD have activated inflammatory cells within these lymphoid tissues capable of producing a cascade of inflammatory responses. Pro‐inflammatory cytokines such as (IL)‐1, IL‐2, and IL‐6 and tumor necrosis factor (TNF)‐α recruit immune cells capable of cross‐reacting with pulmonary mucosal cells, inducing damage to respiratory mucosa [3].

In a registry of 33 patients with IBD, 28 patients developed respiratory symptoms after the diagnosis of IBD. In contrast, the remainder of the patients developed these symptoms post‐colectomy, alluding to the fact that patients with IBD can develop pulmonary symptoms independent of the IBD disease activity and duration of the diagnosis. Making a clear connection between UC and vasculitis‐like pulmonary symptoms can be very challenging as the differentials encompass a broad range of pathologies, especially in the initial phases of the clinical presentation. It is relatively difficult to suspect the association of UC with underlying respiratory comorbidities, especially in those with preexisting chronic pulmonary diseases, history of environmental exposures, residence in endemic areas, as well as those with smoking history. It is equally important to recognize that most of the patients who have had established diagnoses of UC are chronically on Disease‐Modifying Antirheumatic Drugs (DMARDs) to maintain disease remission. Defining the role of exogenous etiologies like DMARDs (Sulfasalazine and Mesalamine) used in the treatment of UC is vital as they have been well studied to cause ILD and other parenchymal lung diseases [4]. Retrospective analysis of patients who are not on high‐risk drug therapy for UC successfully identified the prevalence of pulmonary complications and ILDs, proving a direct association between UC and lung diseases [2]. Delineating the etiological link between ulcerative colitis and pulmonary symptoms thus ultimately proves to be highly challenging amidst a multitude of more plausible risk factors, necessitating meticulous history taking, monitoring symptom progression as well as detailed examination. When unambiguous guidelines are not available for definitive management of complex care like ours, existing literature, as well as an interdisciplinary team approach with a timely expert consultation, can prevent undue delay in diagnostic and therapeutic interventions.

Finally, it is essential to consider that nearly 5% of patients with UC develop PSC [5]. Autoimmune processes leading to the destruction of the biliary tract are the driving factor in the development of PSC [5]. Studies have shown cross‐reactivity between the antigen in the biliary tract and colonic mucosa, which likely explains the strong molecular association between PSC and UC [6]. Antineutrophil cytoplasmic antibodies and antinuclear antibodies are some of the autoantibodies that are shared between these pathologies. The primary modality of diagnosing PSC is ERCP, which generally reveals multiple segments of strictures with normal anatomy in between, giving it a “beads on string” appearance. Histologically, periductal fibrosis leads to onion skin appearance [7]. Nearly 5% of patients with UC develop PSC, which is associated with devastating complications of advanced liver fibrosis, primary hepatocellular carcinoma, and cholangiocarcinoma [5]. Adenocarcinoma of the biliary ducts progresses rapidly, with an average survival rate of about 6 months after diagnosis [8]. Intraperitoneal seeding is standard, leading to metastatic disease [5]. Given its association with significant complications, it is imperative to maintain a high level of suspicion and ongoing monitoring for the development of PSC in patients with UC. Similarly, patients with PSC have an increased risk of developing UC. All patients with PSC need to be evaluated with a colonoscopy at the time of PSC diagnosis to rule out UC. Some of the characteristic features, including rectal sparing, pancolitis, and backwash ileitis, are seen in PSC‐associated UC, which carries a higher risk for colon cancer and needs closer surveillance with colonoscopy and biopsies every year from the time of diagnosis [9].

Finally, a key piece of information that can be extrapolated from our case is issues related to cognitive bias. In our emphasis on the patient's initial pulmonary manifestations, we inadvertently delayed the diagnosis of other serious conditions like UC and PSC. The existence of such “tunnel vision” bias in medicine is not uncommon, especially when managing complicated conditions with multiorgan involvement. Mitigation requires comprehensive consideration of diverse clinical presentations and concurrent conditions to prevent undue disease progression and life‐threatening consequences.

## 6 Conclusion

In summary, this is a case of a patient diagnosed with interstitial lung disease (ILD), initially believed to be due to vasculitis, who was discovered to have UC with coexisting PSC. ILDs, if diagnosed, often manifest during the disease course or are attributed to medications used for UC. This case illustrates the diagnostic complexity posed due to the atypical presentation of UC reflecting an anchoring bias. Early recognition of the condition is crucial to tailor an appropriate treatment regime targeting multiple coexisting pathologies and thus preventing undue overlapping therapies with potentially severe adverse effects. It is equally important to highlight that nearly 5% of patients with UC develop PSC, which is associated with devastating complications of advanced liver fibrosis, primary hepatocellular carcinoma, and cholangiocarcinoma. Although routine screening of PSC is not recommended, patients with UC with abnormal liver tests need immediate evaluation for PSC and further diagnostic modalities. A timely diagnosis is crucial, given the high risk associated with the condition. As the evaluation and diagnosis of IBD‐associated lung parenchymal disease can be very challenging, early recognition is of utmost importance.

## Author Contributions

**Pritee Shrestha:** conceptualization, data curation, formal analysis, investigation, methodology, project administration, resources, supervision, writing – original draft, writing – review and editing. **Philip Shara:** data curation, investigation, methodology, validation, writing – review and editing. **Babajide Adio:** conceptualization, data curation, formal analysis, investigation, methodology, project administration, resources, supervision, validation, writing – review and editing.

## Consent

Patient has signed the consent form for this case report.

## Data Availability

The authors have nothing to report.
